# Supplementary material for: Evolutionary Study of Disorder in Protein Sequences
Source: Biomolecules. 2020 Oct 6;10(10):1413. doi: 10.3390/biom10101413 (PMC7650552; doi:10.3390/biom10101413)
Supplement: Supplementary file 1 [file biomolecules-10-01413-s001.zip › biomolecules-936875-supplementary v5/biomolecules-936875-supplementary.docx]

Supplementary figures


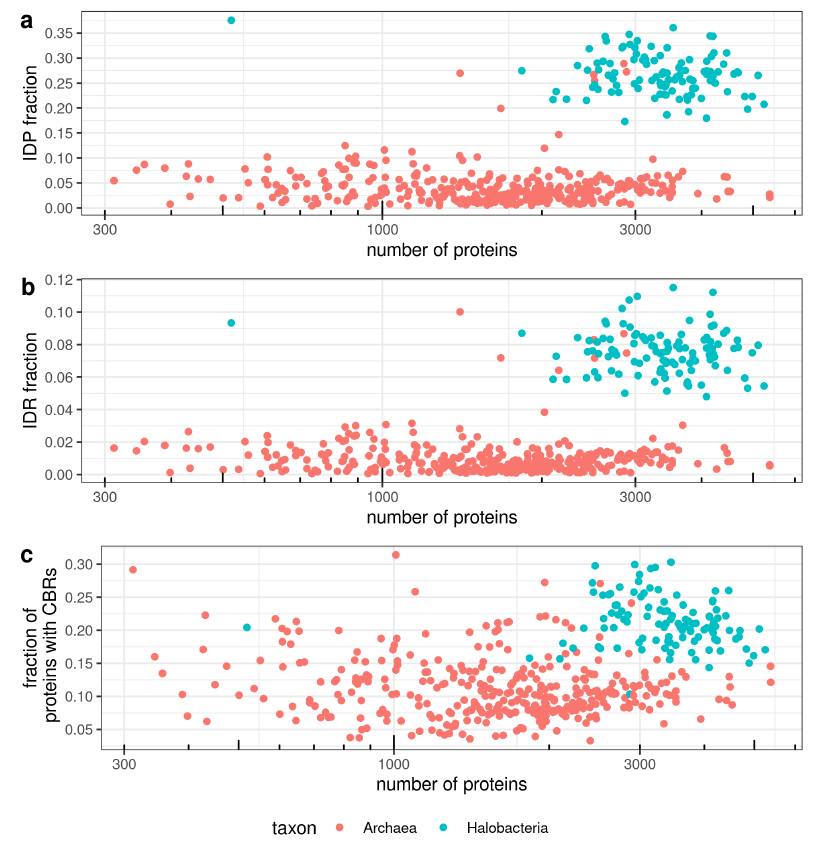


**Figure S1.** Disorder and CBR content in Halobacteria and other Archaea**.** (**a**) IDP fraction (**b**) IDR fraction and (**c**) fraction of proteins with CBRs vs. number of proteins.


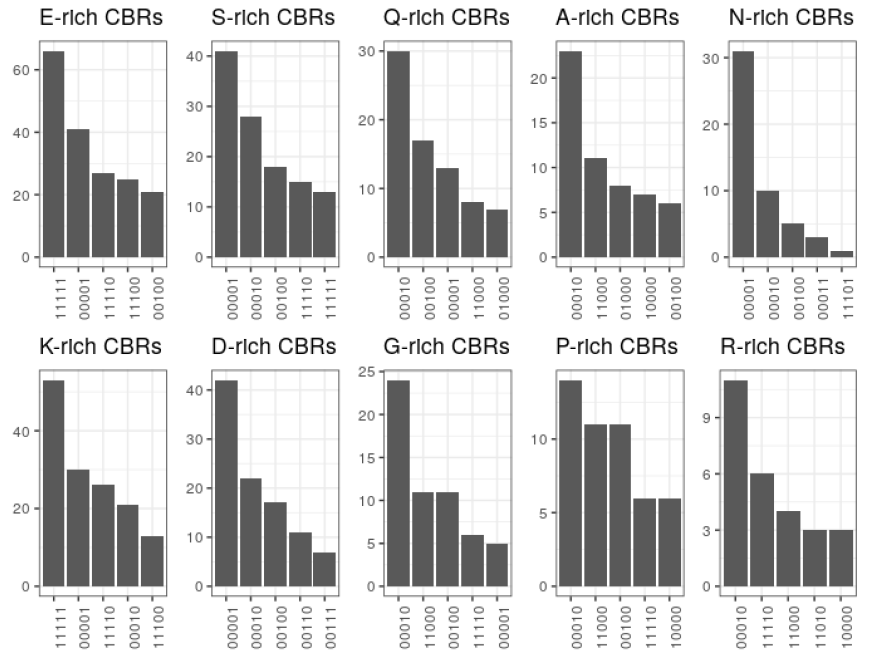


**Figure S2.** Five most frequent conservation patterns for the 10 most frequent X-rich CBRs**.**

Supplementary Table Captions:

**Supplementary** **Table S1.** Measurements of disorder and composition bias for different species. Columns indicate values of disorder for a given species.

**Supplementary Table S2.** Abbreviations of species names used in Figure 3.

**Supplementary** **Table S3.** Groups of orthologs used. Columns indicate clusters (as displayed in Figure 5), and Jaccard scores indicating similarity in disorder position to the human ortholog. Identifiers are UniProt ACs (accession numbers).

**Supplementary** **Table S4.** Results of gene functional enrichment analysis for human disordered proteins. Columns indicate cluster, GO term category, number of genes with the term in the background, number of genes with the term in the cluster, expected number of genes, fold of enrichment, p-value of enrichment, list of UniProt IDs.

**Supplementary** **Table S5.** Results of gene functional enrichment analysis for *D. melanogaster* orthologs to human IDPs. Columns as in Suppl. Table S4.

**Supplementary** **Table S6.** Results of gene functional enrichment analysis for *S. cerevisiae* orthologs to human IDPs. Columns as in Suppl. Table S4.

**Supplementary** **Table S7.** Frequency of CBR conservation patterns.
